# Changes in adiposity over the life course and gene expression in postmenopausal women

**DOI:** 10.1002/cam4.4649

**Published:** 2022-03-19

**Authors:** Yunan Han, Graham A. Colditz, Adetunji T. Toriola

**Affiliations:** ^1^ Division of Public Health Sciences, Department of Surgery Washington University School of Medicine Saint Louis Missouri USA; ^2^ Alvin J. Siteman Cancer Center Barnes‐Jewish Hospital and Washington University School of Medicine Saint Louis Missouri USA

**Keywords:** adiposity, BMI change, gene expression, growth factor, postmenopausal, RANK pathway, sex hormone

## Abstract

**Background:**

Early life adiposity and changes in adiposity over the life course are associated with mammographic breast density among postmenopausal women. However, the underlying mechanisms are unknown; therefore, we comprehensively examined the associations of early life body mass index (BMI) and changes in BMI from ages 10, 18 to age at mammogram with growth factor, RANK pathway, and sex hormone gene expression in 372 postmenopausal women.

**Methods:**

We estimated early life BMI at age 10 using the validated 9‐level Stunkard pictogram. We calculated BMI at other ages (18, 30, and current age at mammogram) by dividing weight in kilograms at these ages with height in meters squared. Sequencing for gene expression was performed using the NanoString nCounter system. After adjusting for confounders, we estimated associations using multivariable linear regressions.

**Results:**

A 10 kg/m^2^ increase in early life BMI at age 10 was associated with a 17.2% decrease in RANKL gene expression (95% confidence interval [CI] = −30.8, −0.9) but was not associated with changes in other markers. BMI changes from ages 10, 18 to age at mammogram were associated with an increase in BMP2 and decreases in RANK, RANKL, and TNFRSF13B gene expression but were not associated with gene expression of other markers. A 10 kg/m^2^ increase in early life BMI from age 10 to current age was associated with a 7.8% increase in BMP2 (95% CI = −1.4, 17.8), an 8.5% decrease in RANK (95% CI = −13.9, −2.8), a 10.4% decrease in RANKL (95% CI = −16.9, −3.3), and an 8.5% decrease in TNFRSF13B gene expression (95% CI = −13.8, −2.8).

**Conclusion:**

The results provide new insights into the biological mechanisms underlying the associations of adiposity changes from early life to adulthood and early life adiposity with mammographic breast density in postmenopausal women.

## Graphical abstract

We examined the associations of early‐life body mass index (BMI) and changes in BMI from ages 10, 18 to age at mammogram with growth factor, RANK pathway, and sex hormone gene expression in 372 postmenopausal women. Our study shows that changes in BMI from childhood, late adolescence, and early adulthood were associated with BMP2, RANK, RANKL, and TNFRSF13B gene expression in postmenopausal women.

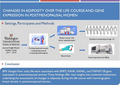

## 1 INTRODUCTION

Obesity is a global public health problem. From 1999–2000 to 2017–2018, US obesity prevalence increased from 30.5% to 42.4%.^1^ Excess adiposity tends to accrue during early and middle adulthood.^2^ Among US adults, the mean weight gain is 0.5–1.0 kg/year during early and middle adulthood.^3^ Although modest, this yearly accumulation of weight eventually leads to obesity over time.^2^ Excess adiposity is a well‐established risk factor for major chronic diseases, certain types of cancer such as postmenopausal breast cancer,^4^, ^5^ and premature death.^6^ Furthermore, we and others have demonstrated that early life adiposity and long‐term adiposity gains from ages 10 and 18 are associated with breast cancer and mammographic breast density among postmenopausal women.^7^, ^8^, ^9^, ^10^, ^11^ However, the underlying biological mechanisms driving the associations of early life adiposity with mammographic breast density and breast cancer are largely unknown.

Growth factors regulate several physiological functions related to growth, development, and glucose homeostasis.^12^ Insulin‐like growth factors (IGFs) and their binding proteins, insulin‐like growth factor‐binding proteins (IGFBPs), are critical modulators of metabolism and data suggest that IGF‐1 and IGFBP‐3 may be associated with mammographic breast density.^13^, ^14^, ^15^, ^16^, ^17^ Early life adiposity is inversely associated with the circulating levels of IGF‐1,^18^, ^19^, ^20^ thus, changes in IGF‐1 levels over time may represent a potential link between early life adiposity and mammographic breast density later in life. Other biomarkers such as sex hormones and the receptor activator of nuclear factor‐kappa (RANK) are associated with mammographic breast density and breast cancer.^21^, ^22^, ^23^, ^24^, ^25^, ^26^, ^27^ Thus, changes in adiposity over time may be associated with changes in these markers. To the best of our knowledge, there are no data on how changes in adiposity over time may be associated with these biomarkers. We, thus, investigated the associations of early life body mass index (BMI) at ages 10 and 18 as well as subsequent changes in BMI from ages 10, 18 to adulthood with growth factor, sex hormone, and RANK pathway gene expression in postmenopausal women. Unraveling these associations will help further expand our knowledge of how early life adiposity and adiposity changes over the life course impact mammographic breast density and breast cancer risk.

## 2 METHODS

### 2.1 Study design and participants

We recruited 400 postmenopausal women who were scheduled for annual screening mammograms at Washington University School of Medicine (Joanne Knight Breast Health Center, Siteman Cancer Center) from October 2017 to September 2018. A detailed description of the study participants has been presented in our previous paper.^7^ In brief, the inclusion criteria were (a) women aged 50–64 years, (b) postmenopausal women, (c) able to comply with requirements according to all procedures and schedules, including the collection of blood samples at enrollment.^7^ The exclusion criteria were (a) any cancer history, including breast cancer; (b) history of breast implants, augmentation, or reduction; and (c) use of selective estrogen receptor modulators (SERM) or denosumab in the previous 6 months.^7^

For 2–4 weeks before the scheduled mammograms, research coordinators mailed research flyers to eligible participants. On the day of the appointment, each study participant completed a blood draw by trained phlebotomists and filled in breast cancer risk factor questionnaires.^7^ We excluded 16 women with missing gene expression data and 12 women with missing BMI or body shape at age 10; thus, we had 372 postmenopausal women in our final analysis. This study obtained institutional review board (IRB) approval from Washington University School of Medicine, and documented consent was obtained from all participants.

### 2.2 BMI measures and BMI change

Body shape at age 10 was measured with the 9‐level figure somatotype pictogram, which was developed by Stunkard and colleagues.^28^ Then, we estimated BMI at age 10 using the Growing Up Today Study.^7^, ^29^ Since the 9‐level figure somatotype pictogram for girls in the Growing Up Today Study ranged from 1 to 7, we did not compute BMI at age 10 for women whose body shape at age 10 was larger than 7. We calculated BMI at ages 18, 30, and at the time of mammogram by dividing weight in kilograms at each age with height in meters squared. Height was measured using a fixed stadiometer. Weights at ages 18 and 30 were obtained from questionnaires provided by study participants. Weight at mammogram was measured in light clothing without shoes using the full‐body sensing, comprehensive body composition monitor (OMRON, model HBF‐514C).

We defined three initial ages (ages 10, 18, and 30) for BMI change. We derived the following BMI change trajectories for three time periods: (a) from age 10 to age at mammogram, (b) from age 18 to age at mammogram, and (c) from age 30 to age at mammogram.^7^ We defined the categories of BMI change as follows: (a) BMI loss, (b) BMI gain of 0.1–5 kg/m^2^, (c) BMI gain of 5.1–10 kg/m^2^, (d) BMI gain of 10.1–15 kg/m^2^, and (e) BMI gain of >15 kg/m^2^.

### 2.3 Gene expression

We quantified gene expression of growth factors, RANK pathway markers, and sex hormones from participants’ plasma samples. We sequenced the following genes based on a priori hypothesis that these markers may be associated with mammary epithelial and/or stromal proliferation, hence, may be associated with mammographic breast density and breast cancer development. These genes include (I) growth factors: bone morphogenetic protein 2 (BMP2), IGF‐1, IGFBP‐3, fibroblast growth factor 1 (FGF1), fibroblast growth factor 12 (FGF12), and transforming growth factor‐beta 1 (TGFB1); (II) RANK pathway: RANK, RANK ligand (RANKL), tumor necrosis factor receptor superfamily member 13B (TNFRSF13B), tumor necrosis factor receptor superfamily member 18 (TNFRSF18), and osteoprotegerin (OPG); (III) sex hormones: prolactin (PRL), progesterone receptor (PGR), estrogen receptor 1 (ESR1), signal transducer and activator of transcription 1 (STAT1), and signal transducer and activator of transcription 5 (STAT5).

Sequencing for gene expression was performed at the McDonnell Genome Institute, Washington University School of Medicine (WUSM) in Saint Louis. Gene expression was measured in RNA isolated from plasma, using the NanoString nCounter XT Codeset Gene Expression Assays protocol (NanoString Technologies). Samples were processed according to the manufacturer’s recommendations. Hybridization of the RNA to the custom XT Codeset was performed with the inputs of 100 ng (18 samples), 180 ng (1 sample), and 200 ng (384 samples). Following hybridization, samples were processed on the NanoString Prep Station, where they were purified and immobilized on a sample cartridge for data collection. The output for each sample was imported into nSolver Analysis Software for Quality Control and analysis. Binding densities ranged from 0.09 to 0.34. Digital transcript counts from the NanoString nCounter assay were normalized using several housekeeping genes including ACTB, RPLP0, and SF3A1.

### 2.4 Statistical analysis

We calculated means with standard deviations (SD) for continuous variables and percentages (%) for categorical variables. The analysis of variance and Chi‐squared tests were used to compare baseline continuous and categorical variables by BMI change from age 10 to current age at mammogram. We used multivariable‐adjusted linear regressions to evaluate the associations between BMI measures and gene expression, adjusting for age at mammogram, race, family history of breast cancer, and menopausal hormone therapy. First, we analyzed the association between early life BMI (ages 10 and 18) and gene expression. We used BMI per 10 kg/m^2^ to show the magnitude of association for BMI at age 10 as a continuous variable. Next, we analyzed the associations between BMI changes (continuous and categorical) for three time intervals and gene expression. The multivariable‐adjusted models were adjusted for confounders mentioned above, as well as BMI at age 10, to remove any residual confounding variables that may arise from the participants’ starting BMI. For BMI change as a continuous variable, we used BMI change per 10 kg/m^2^ to report the magnitude of association. For BMI change as a categorical variable, we additionally report *p* values for linear trends. Due to the open‐ended categories, we calculated the median values of every category and used appropriate orthogonal polynomial coefficients for trend analyses.^30^ Gene expression levels were all log_2_‐transformed to ensure the normality of the residuals in statistical models. We back‐transformed the beta coefficients (*β*) and 95% confidence intervals (CIs) from the regression models to make interpretation easier. The back‐transformed β was presented as percentage differences (Diff); estimated as Diff% = (exp_2_(*β*)−1)*100 and interpreted as the one‐unit change in an adiposity measure associated with percent change in gene expression. We further assessed the interactions between variables (race and family history of breast cancer) and BMI change over the life course (continuous) by including cross‐product terms (i.e., BMI change × race, BMI change × family history of breast cancer) in multivariable‐adjusted models. We performed the analyses through SAS statistical software (version 9.4). *p* values of <0.05 were considered statistically significant, and all *p* values were two‐sided.

## 3 RESULTS

### 3.1 Participant characteristics

The mean age at the time of mammogram was 58 years (range, 50–65 years, Table 1). More than half (61.0%) of the participants were non‐Hispanic Whites and 36.3% were African Americans. The majority (50.3%) had body shape 1–2 at age 10. Nineteen women (5.1%) had a BMI gain of 0.1–5 kg/m^2^ from age 10 to age at mammogram, 102 (27.4%) had a BMI gain of 5.1–10 kg/m^2^, 109 (29.3%) had a BMI gain of 10.1–15 kg/m^2^, and 141 (37.9%) had a BMI gain of >15 kg/m^2^, whereas only one participant had a BMI loss. Women who had a BMI gain of >15 kg/m^2^ from age 10 to age at mammogram were more likely to be African Americans (44.7%) and less likely to use menopausal hormone therapy (28.4%) (Table 1).

**TABLE 1:** Characteristics of 372 postmenopausal women by BMI change from age 10 to age at mammogram, recruited during annual screening mammogram at the Joanne Knight Breast Health Center, Washington University School of Medicine, St. Louis, MO

Characteristics	Total^a^		BMI gain of 0.1–5 kg/m^2^		BMI gain of 5.1–10 kg/m^2^		BMI gain of 10.1–15 kg/m^2^		BMI gain of >15 kg/m^2^		*p* value^c^
	*N*	Mean ± SD/Percentage (%)^b^	*N*	Mean ± SD/Percentage (%)^b^	*N*	Mean ± SD/Percentage (%)^b^	*N*	Mean ± SD/Percentage (%)^b^	*N*	Mean ± SD/Percentage (%)^b^	
Age (years)	372	58.0 ± 3.9	19	59.6 ± 3.8	102	57.8 ± 3.7	109	58.0 ± 4.0	141	57.9 ± 3.8	0.28
Race											**<0.001**
Non‐Hispanic White	227	61.0%	14	73.7%	70	68.6%	67	61.5%	76	53.9%	
African American	135	36.3%	5	26.3%	28	27.5%	39	35.8%	63	44.7%	
Others	10	2.7%	—	—	4	3.9%	3	2.8%	2	1.4%	
Ever used menopausal hormone therapy											**0.02**
Yes	123	33.1%	12	63.2%	31	30.4%	39	35.8%	40	28.4%	
No	249	66.9%	7	36.8%	71	69.6%	70	64.2%	101	71.6%	
Family history of breast cancer^d^											
Yes	92	24.7%	6	31.6%	24	23.5%	25	22.9%	36	25.5%	0.81
No	275	73.9%	13	68.4%	76	74.5%	83	76.2%	103	73.1%	
Adiposity measures^e^											
BMI at age 10 years (kg/m^2^)	372	17.2 ± 3.0	19	18.8 ± 3.2	102	17.1 ± 2.9	109	17.1 ± 3.0	141	17.2 ± 3.0	0.19
BMI at age 18 years (kg/m^2^)	372	21.8 ± 4.5	19	20.0 ± 2.2	102	20.6 ± 3.4	109	21.3 ± 4.0	141	23.2 ± 5.3	**<0.001**
BMI at age 30 years (kg/m^2^)	372	25.1 ± 5.9	19	21.4 ± 2.6	102	22.5 ± 3.8	109	24.0 ± 4.1	141	28.4 ± 7.1	**<0.001**
BMI at mammogram (kg/m^2^)	372	31.3 ± 7.7	19	22.1 ± 3.7	102	24.8 ± 3.2	109	29.8 ± 3.3	141	38.5 ± 6.4	**<0.001**
Body shape at age 10 years											0.12
1–2	187	50.3%	4	21.1%	51	50.0%	62	56.9%	70	49.7%	
3–4	111	29.8%	9	47.3%	32	31.4%	26	23.9%	44	31.2%	
5	42	11.3%	3	15.8%	10	9.8%	11	10.1%	17	12.1%	
6+	32	8.6%	3	15.8%	9	8.8%	10	9.2%	10	7.1%	

Abbreviations: BMI, body mass index; SD, standard deviation.

^a^ One case was BMI loss from age 10 to age at mammogram.

^b^ We presented column percentage (%).

^c^ *p*‐values were calculated for a comparison across all groups by BMI change from age 10 to age at mammogram from Chi‐square tests, and analysis of variance (ANOVA) tests as appropriate.

^d^ Five women had missing data for family history of breast cancer.

^e^ Body mass index (BMI) was calculated as weight (kg) at each age divided by height squared (m^2^).

Bold indicates *P*‐values less than 0.05 were considered statistically significant, and all *P*‐values were two‐sided.

### 3.2 Early life BMI and gene expression

BMI at age 10 was inversely associated with RANKL gene expression after controlling for age at mammogram, race, family history of breast cancer, and menopausal hormone therapy use (Table 2 **)**. A 10 kg/m^2^ increase in BMI at age 10 was associated with a 17.2% decrease in RANKL gene expression (95% CI = −30.8, −0.9). BMI at age 10 was not associated with gene expression of other RANK pathway markers, growth factors, and sex hormones evaluated in this analysis. BMI at age 18 was not associated with changes in profiled gene expression (Table S1).

**TABLE 2:** Multivariable‐adjusted associations between BMI at age 10 and plasma gene expression profile in 372 postmenopausal women^a^

Genes^b^	Per 10 kg/m^2^ BMI increase at age 10	
	Diff%^c^	95% CI
Growth factor‐related genes		
BMP2	−1.3	−20.0, 21.6
IGF‐1	−9.6	−29.9, 16.6
IGFBP‐3	−2.3	−17.2, 15.4
FGF1	2.5	−5.5, 11.1
FGF12	−0.1	−8.0, 8.5
TGFB1	0.4	−7.8, 9.4
RANK pathway‐related genes		
RANK	−7.5	−19.9, 6.7
RANKL	−**17.2** *	**−30.8, −0.9**
TNFRSF13B	−11.1	−22.8, 2.4
TNFRSF18	1.5	−7.1, 10.8
OPG	−4.2	−15.8, 9.1
Sex hormone‐related genes		
PRL	−4.3	−11.5, 3.4
PGR	0.3	−11.5, 13.7
ESR1	−3.9	−12.9, 6.1
STAT1	10.3	−3.8, 26.6
STAT5	3.7	−5.2, 13.3

Abbreviations: BMI, body mass index; CI, confidence interval.

^a^ Multivariable‐adjusted models were adjusted for age at mammogram (continuous, years), race (non‐Hispanic white/African American/others), family history of breast cancer (yes/no/unknown), and menopausal hormone therapy (yes/no).

^b^ Gene expression was presented as the mean and standard deviation of log_2_‐transformed values.

^c^ Diff% represents the one‐unit change in an adiposity measure associated with a % change in gene expression.

* *p* value = 0.04.

Bold indicates *P*‐values less than 0.05 were considered statistically significant, and all *P*‐values were two‐sided.

### 3.3 BMI change and growth factor gene expression

After controlling for BMI at age 10 and above confounders, increases in BMI from ages 10 and 18 to current age were associated with an increase in BMP2 gene expression, but there were no associations with other growth factors (Table 3). A 10 kg/m^2^ increase in BMI from age 10 was associated with a 7.8% increase in BMP2 gene expression (95% CI = −1.4, 17.8). Compared with women who had a BMI gain of 0.1–5 kg/m^2^ from age 10, a 5.1–10 kg/m^2^ BMI gain was associated with a 39.3% increase (95% CI = 2.7–89.1); a 10.1–15 kg/m^2^ gain was associated with a 38.5% increase (95%CI = 2.4–87.4); and a >15 kg/m^2^ gain was associated with a 50.0% increase (95% CI = 11.2–102.2) in BMP2 gene expression (*p* trend = 0.01) (Table 3).

**TABLE 3:** Multivariable‐adjusted associations between BMI change from ages 10 and 18 (continuous and categorical) to age at mammogram and plasma growth factor gene expression profile in 372 postmenopausal women^a^

Genes^b^	BMI change (continuous)	BMI change (categories)					
	Per 10 kg/m^2^ BMI increase, Diff% (95% CI)^c^	BMI loss, Diff% (95% CI)^c^	BMI gain of 0.1–5 kg/m^2^, Diff% (95% CI)^c^	BMI gain of 5.1–10 kg/m^2^, Diff% (95% CI)^c^	BMI gain of 10.1–15 kg/m^2^, Diff% (95% CI)^c^	BMI gain of >15 kg/m^2^, Diff% (95% CI)^c^	*p* trend
BMP2							
BMI change from age 10 to age at mammogram (kg/m^2^)	7.8 (−1.4, 17.8)	—	Ref.	**39.3 (2.7, 89.1)**	**38.5 (2.4, 87.4)**	**50.0 (11.2, 102.2)**	**0.01**
BMI change from age 18 to age at mammogram (kg/m^2^)	**12.6 (2.5, 23.7)**	5.6 (−22.9, 44.5)	Ref.	**25.5 (5.0, 50.1)**	**24.9 (4.1, 49.8)**	**34.8 (10.0, 65.1)**	**0.02**
IGF‐1							
BMI change from age 10 to age at mammogram (kg/m^2^)	−5.8 (−15.5, 5.0)	—	Ref.	−7.2 (−36.2, 34.9)	−17.7 (−43.1, 19.2)	−14.5 (−40.7, 23.3)	0.82
BMI change from age 18 to age at mammogram (kg/m^2^)	−5.9 (−16.1, 5.6)	37.0 (−6.8, 101.4)	Ref.	14.8 (−7.8, 43.0)	0.2 (−19.8, 25.3)	−0.2 (−22.2, 28.0)	0.09
IGFBP‐3							
BMI change from age 10 to age at mammogram (kg/m^2^)	−2.3 (−9.0, 4.8)	—	Ref.	−7.3 (−27.3, 18.3)	−9.8 (−29.1, 14.8)	−10.7 (−29.6, 13.4)	0.82
BMI change from age 18 to age at mammogram (kg/m^2^)	−2.2 (−9.3, 5.4)	20.9 (−6.0, 55.4)	Ref.	−4.5 (−17.2, 10.1)	0.6 (−13.0, 16.4)	−1.9 (−16.6, 15.4)	0.26
FGF1							
BMI change from age 10 to age at mammogram (kg/m^2^)	−0.3 (−3.7, 3.2)	—	Ref.	**14.0 (1.2, 28.4)**	**13.1 (0.5, 27.2)**	12.1 (−0.2, 25.9)	**0.03**
BMI change from age 18 to age at mammogram (kg/m^2^)	−1.0 (−4.6, 2.7)	−1.7 (−13.0, 11.0)	Ref.	**7.7 (0.5, 15.5)**	0.9 (−6.0, 8.3)	−0.4 (−8.0, 7.8)	**0.05**
FGF12							
BMI change from age 10 to age at mammogram (kg/m^2^)	−0.1 (−3.6, 3.4)	—	Ref.	0.9 (−10.6, 13.9)	−3.2 (−14.1, 9.2)	−0.1 (−11.2, 12.5)	0.39
BMI change from age 18 to age at mammogram (kg/m^2^)	−0.9 (−4.5, 2.9)	3.1 (−9.0, 16.8)	Ref.	6.9 (−0.5, 14.7)	1.7 (−5.4, 9.4)	3.7 (−4.4, 12.4)	0.28
TGFB1							
BMI change from age 10 to age at mammogram (kg/m^2^)	0.8 (−2.8, 4.6)	—	Ref.	−8.2 (−19.0, 4.1)	−3.8 (−15.0, 8.9)	−3.7 (−14.8, 8.8)	0.15
BMI change from age 18 to age at mammogram (kg/m^2^)	−1.2 (−4.9, 2.7)	−1.2 (−13.2, 12.5)	Ref.	−1.0 (−8.0, 6.6)	1.8 (−5.6, 9.7)	−2.4 (−10.3, 6.1)	0.98

Abbreviations: BMI, body mass index; CI, confidence interval.

^a^ Multivariable‐adjusted models were adjusted for age at mammogram (continuous, years), BMI at age 10 (continuous, kg/m^2^), race (non‐Hispanic white/African American/others), family history of breast cancer (yes/no/unknown), and menopausal hormone therapy (yes/no).

^b^ Gene expression was presented as the mean and standard deviation of log_2_‐transformed values.

^c^ Diff% represents the one‐unit change in an adiposity measure associated with a % change in gene expression.

Bold indicates *P*‐values less than 0.05 were considered statistically significant, and all *P*‐values were two‐sided.

### 3.4 BMI change and RANK pathway gene expression

Increases in BMI from ages 10 and 18 to current age were associated with decreases in RANK, RANKL, and TNFRSF13B gene expression but not TNFRSF18 and OPG gene expression (Table 4). A 10 kg/m^2^ increase in BMI from age 10 was associated with an 8.5% decrease in RANK (95% CI = −13.9 to −2.8), a 10.4% decrease in RANKL (95% CI = −16.9 to −3.3), and an 8.5% decrease in TNFRSF13B gene expression (95%CI = −13.8 to −2.8). Compared with women who had a BMI gain of 0.1–5 kg/m^2^ from age 10, a 5.1–10 kg/m^2^ BMI gain was associated with a 13.5% decrease (95% CI = −29.8 to 6.6); a 10.1–15 kg/m^2^ gain was associated with an 18.5% decrease (95% CI = −33.7 to 0.2); and a >15 kg/m^2^ gain was associated with a 22.5% decrease (95% CI = −36.9 to −5.0) in RANK gene expression (*p* trend = 0.01). Similar associations were observed for RANKL and TNFRSF13B gene expression (Table 4).

**TABLE 4:** Multivariable‐adjusted associations between BMI change from ages 10 and 18 (continuous and categorical) to age at mammogram and plasma RANK pathway‐related genes expression profile in 372 postmenopausal women^a^

Genes^b^	BMI change (continuous)	BMI change (categories)					
	Per 10 kg/m^2^ BMI increase, Diff% (95% CI)^c^	BMI loss, Diff% (95% CI)^c^	BMI gain of 0.1–5 kg/m^2^, Diff% (95% CI)^c^	BMI gain of 5.1–10 kg/m^2^, Diff% (95% CI)^c^	BMI gain of 10.1–15 kg/m^2^, Diff% (95% CI)^c^	BMI gain of >15 kg/m^2^, Diff% (95% CI)^c^	*p* trend
RANK							
BMI change from age 10 to age at mammogram (kg/m^2^)	**−8.5 (−13.9, −2.8)**	—	Ref.	−13.5 (−29.8, 6.6)	−18.5 (−33.7, 0.2)	**−22.5 (−36.9, −5.0)**	**0.01**
BMI change from age 18 to age at mammogram (kg/m^2^)	**−8.9 (−14.6, −2.8)**	−0.8 (−20.1, 23.0)	Ref.	−8.8 (−19.3, 3.1)	−7.9 (−18.7, 4.4)	**−19.5 (−30.0, −7.5)**	**0.003**
RANKL							
BMI change from age 10 to age at mammogram (kg/m^2^)	**−10.4 (−16.9, −3.3)**	—	Ref.	9.1 (−16.1, 42.0)	−7.0 (−28.3, 20.7)	−5.8 (−27.2, 21.8)	0.44
BMI change from age 18 to age at mammogram (kg/m^2^)	**−11.9 (−18.8, −4.6)**	2.6 (−21.8, 34.5)	Ref.	−8.4 (−21.5, 6.8)	−9.7 (−22.9, 5.6)	**−21.1 (−33.8, −6.0)**	**0.02**
TNFRSF13B							
BMI change from age 10 to age at mammogram (kg/m^2^)	**−8.5 (−13.8, −2.8)**	—	Ref.	−13.7 (−29.8, 6.0)	**−21.3 (−35.8, −3.5)**	**−22.8 (−36.9, −5.5)**	**0.01**
BMI change from age 18 to age at mammogram (kg/m^2^)	**−9.5 (−15.0, −3.6)**	20.7 (−2.4, 49.2)	Ref.	−4.4 (−15.2, 7.9)	**−11.7 (−21.9, −0.2)**	−10.7 (−22.2, 2.4)	**0.006**
TNFRSF18							
BMI change from age 10 to age at mammogram (kg/m^2^)	−2.7 (−6.3, 0.9)	—	Ref.	−6.1 (−17.5, 6.8)	−7.1 (−18.3, 5.5)	−8.0 (−18.9, 4.4)	0.21
BMI change from age 18 to age at mammogram (kg/m^2^)	−2.7 (−6.5, 1.3)	−0.8 (−13.1, 13.4)	Ref.	−3.3 (−10.4, 4.3)	−1.1 (−8.4, 6.8)	−7.3 (−14.9, 1.0)	0.36
OPG							
BMI change from age 10 to age at mammogram (kg/m^2^)	−2.7 (−7.9, 2.9)	—	Ref.	−9.1 (−24.8, 10.0)	−10.1 (−25.5, 8.6)	−9.0 (−24.4, 9.7)	0.32
BMI change from age 18 to age at mammogram (kg/m^2^)	−2.6 (−8.1, 3.3)	4.8 (−13.9, 27.7)	Ref.	1.4 (−9.4, 13.4)	−2.9 (−13.4, 8.8)	−2.1 (−13.8, 11.2)	0.59

Abbreviations: BMI, body mass index; CI, confidence interval.

^a^ Multivariable‐adjusted models were adjusted for age at mammogram (continuous, years), BMI at age 10 (continuous, kg/m^2^), race (non‐Hispanic white/African American/others), family history of breast cancer (yes/no/unknown), and menopausal hormone therapy (yes/no).

^b^ Gene expression was presented as the mean and standard deviation of log_2_‐transformed values.

^c^ Diff% represents the one‐unit change in an adiposity measure associated with a % change in gene expression.

Bold indicates *P*‐values less than 0.05 were considered statistically significant, and all *P*‐values were two‐sided.

### 3.5 BMI change and sex hormone gene expression

We observed no associations between BMI increase (per 10 kg/m^2^ BMI increase) from ages 10 and 18 to the current age and sex hormone gene expressions (Table 5).

**TABLE 5:** Multivariable‐adjusted associations between BMI change from ages 10 and 18 (continuous and categorical) to age at mammogram and plasma sex hormones gene expression profile in 372 postmenopausal women^a^

Genes^b^	BMI change (continuous)	BMI change (categories)					
	Per 10 kg/m^2^ BMI increase, Diff% (95% CI)^c^	BMI loss, Diff% (95% CI)^c^	BMI gain of 0.1–5 kg/m^2^, Diff% (95% CI)^c^	BMI gain of 5.1–10 kg/m^2^, Diff% (95% CI)^c^	BMI gain of 10.1–15 kg/m^2^, Diff% (95% CI)^c^	BMI gain of >15 kg/m^2^, Diff% (95% CI)^c^	*p* trend
PRL							
BMI change from age 10 to age at mammogram (kg/m^2^)	0.5 (−2.8, 3.9)	—	Ref.	6.3 (−5.2, 19.2)	5.2 (−6.0, 17.8)	7.4 (−4.0, 20.1)	0.30
BMI change from age 18 to age at mammogram (kg/m^2^)	1.5 (−2.0, 5.2)	2.5 (−8.9, 15.3)	Ref.	**9.1 (2.0, 16.6)**	4.0 (−2.9, 11.3)	7.4 (−0.5, 15.9)	**0.05**
PGR							
BMI change from age 10 to age at mammogram (kg/m^2^)	−0.6 (−5.7, 4.9)	—	Ref.	−7.7 (−23.2, 10.9)	−6.4 (−22.0, 12.3)	−3.7 (−19.6, 15.2)	0.41
BMI change from age 18 to age at mammogram (kg/m^2^)	−2.1 (−7.5, 3.6)	3.5 (−14.5, 25.2)	Ref.	−2.2 (−12.2, 9.0)	−1.2 (−11.5, 10.4)	0.8 (−10.9, 14.0)	0.97
ESR1							
BMI change from age 10 to age at mammogram (kg/m^2^)	−0.5 (−4.6, 3.7)	—	Ref.	−11.1 (−23.1, 2.7)	−11.4 (−23.2, 2.2)	−9.1 (−21.1, 4.7)	0.77
BMI change from age 18 to age at mammogram (kg/m^2^)	−2.2 (−6.5, 2.3)	1.4 (−12.8, 17.8)	Ref.	−3.0 (−11.0, 5.6)	−1.0 (−9.2, 8.0)	−3.6 (−12.5, 6.2)	0.45
STAT1							
BMI change from age 10 to age at mammogram (kg/m^2^)	−4.3 (−9.7, 1.4)	—	Ref.	1.7 (−16.9, 24.4)	−6.0 (−23.0, 14.8)	−6.4 (−23.2, 14.0)	0.21
BMI change from age 18 to age at mammogram (kg/m^2^)	−5.0 (−10.7, 1.1)	3.5 (−16.0, 27.4)	Ref.	4.7 (−7.0, 17.8)	−3.8 (−14.7, 8.5)	−7.5 (−19.1, 5.8)	0.23
STAT5							
BMI change from age 10 to age at mammogram (kg/m^2^)	0.7 (−3.0, 4.6)	—	Ref	4.4 (−8.4, 19.0)	10.2 (−3.2, 25.5)	6.6 (−6.2, 21.2)	0.16
BMI change from age 18 to age at mammogram (kg/m^2^)	0.9 (−3.1, 5.1)	0.5 (−12.1, 14.9)	Ref.	**11.6 (3.4, 20.4)**	6.9 (−1.1, 15.6)	4.4 (−4.3, 13.9)	**0.04**

Abbreviations: BMI, body mass index; CI, confidence interval.

^a^ Multivariable‐adjusted models were adjusted for age at mammogram (continuous, years), BMI at age 10 (continuous, kg/m^2^), race (non‐Hispanic white/African American/others), family history of breast cancer (yes/no/unknown), and menopausal hormone therapy (yes/no).

^b^ Gene expression was presented as the mean and standard deviation of log_2_‐transformed values.

^c^ Diff% represents the one‐unit change in an adiposity measure associated with a % change in gene expression.

Bold indicates *P*‐values less than 0.05 were considered statistically significant, and all *P*‐values were two‐sided.

### 3.6 Tests for interaction

We evaluated interactions of BMI change over the life course with race and family history of breast cancer. We observed interactions of TGFB1 and IGFBP‐3 gene expression with a family history of breast cancer (data not shown). When stratified by family history of breast cancer, we observed that a 10 kg/m^2^ increase in BMI from age 10 was associated with a 6.6% increase in TGFB1 gene expression among women with a positive family history of breast cancer and a 0.8% decrease among women with no family history of breast cancer (*p*_interaction_ = 0.03). A 10 kg/m^2^ increase in BMI from age 18 was associated with a 13.4% increase in IGFBP‐3 gene expression among women with a positive family history of breast cancer and a 7.0% decrease among women with no family history of breast cancer (*p*_interaction_ = 0.02).

Additionally, we reported the results for multivariable‐adjusted associations between BMI change from age 30 years to age at mammogram and gene expression in Table S2.

## 4 DISCUSSION

To the best of our knowledge, this is the first study to investigate the associations of early life BMI and changes in BMI over the life course with gene expression of growth factors, RANK pathway markers, and sex hormones among postmenopausal women. BMI at age 10 was inversely associated with RANKL gene expression, but not with other markers profiled. Increases in BMI from ages 10 and 18 to age at mammogram were associated with increases in BMP2 gene expression but decreases in RANK, RANKL, and TNFRSF13B gene expression. Changes in BMI from ages 10 and 18 were not associated with sex hormone gene expression (ESR1, PGR, PRL, STAT1, and STAT5).

Intriguingly, BMI gain over the life course was positively associated with BMP2 gene expression in postmenopausal women. BMPs are highly conserved functional proteins belonging to the transformation growth TGF‐β superfamily. BMPs were initially identified as inducers of bone and cartilage formation^31^, ^32^ but are now known to signal in adipose tissue and adipogenic differentiation beyond the bone.^33^ BMP2 is expressed at higher levels in visceral than subcutaneous adipose tissue.^34^ A recent genomewide association study (GWAS) meta‐analysis identified a genetic locus annotated to BMP2 (rs979012) that is associated with body fat distribution in women,^35^ further suggesting that BMP2 influences adipose tissue biology. In addition, BMP2 was reported to facilitate epithelial‐to‐mesenchymal transition^36^ and promote the invasiveness of breast cancer cells in vitro and mouse xenograft models.^37^, ^38^ Our findings suggest that BMP2 could be a novel biomarker worth evaluating when investigating the associations of early life adiposity with mammographic breast density in postmenopausal women.

We observed no associations of BMI change over the life course with gene expression of other growth factors (IGF‐1, IGFBP‐3, FGF1, FGF12, and TGFB1). Obese adipose tissue creates a pro‐oncogenic environment that may be associated with increased levels of circulating insulin and IGF‐1.^39^ However, early life adiposity has been shown to be inversely associated with circulating IGF‐1 levels in adults.^18^, ^19^, ^20^ Our findings suggest that the effect of IGF‐1 on mammographic breast density in postmenopausal women is unlikely to be due to the effect of adiposity change over the life course.

BMI gains from ages 10 and 18 to the age at mammogram were inversely associated with RANK, RANKL, and TNFRSF13B gene expression. Obesity may increase bone resorption by upregulating pro‐inflammatory cytokines (IL‐6 and TNF‐α), which can stimulate osteoclast activity through the RANK pathway.^40^, ^41^ RANK and RANKL are members of the TNF superfamily of proteins. The RANK pathway is important in bone homeostasis^42^, ^43^ and immune responses.^44^, ^45^ The RANK pathway also plays an essential role in breast development and hormone‐driven mammary epithelial proliferation.^46^, ^47^, ^48^ We have previously shown that circulating RANK^21^ and RANKL gene expression^22^ were positively associated with mammographic breast density in premenopausal women. Long‐term adiposity gains from ages 10 and 18 were inversely associated with mammographic breast density in postmenopausal women.^7^ Our findings suggest that changes in RANK pathway expression, caused by long‐term adiposity gains, may contribute to the decreased mammographic breast density in postmenopausal women.

Adiposity is positively associated with circulating estrone, estradiol, and testosterone levels and negatively associated with sex hormone‐binding globulin (SHBG) levels, leading to an increase in total bioavailable estrogen in postmenopausal women.^49^, ^50^ Estrogen biosynthesis is catalyzed largely in obese adipose tissue after menopause, through the conversion of adrenal androgens into estrogens by aromatase.^49^ Prepubertal girls (8–10 years old) who were heavy had higher levels of dehydroepiandrosterone sulfate and lower levels of SHBG during puberty compared with those who were lean,^51^ but no study has examined whether adiposity change from childhood and adolescence is associated with sex hormone gene expression. Although we observed no associations between adiposity change over the life course and sex hormone gene expression, larger studies that include other hormones such as SHBG and evaluate potential crosstalk between the hormones are needed.

Epigenetic dysregulation during early development may increase the risk of obesity.^52^ More than 2000 differentially methylated regions in adipose tissue of individuals with unhealthy overweight/obesity versus normal‐weight individuals or individuals with metabolically healthy overweight/obesity have been identified.^53^ However, the epigenetic evidence for long‐term change of obesity from age 10 to postmenopause is lacking. Future studies can integrate genetic and epigenetic data to elucidate the underlying mechanisms driving early life adiposity.

Our study has several strengths. First, we investigated the associations of change in adiposity with gene expression, rather than circulating proteins since protein quantification still lags behind the high‐throughput experimental techniques used to determine mRNA expression levels.^54^ Compared with circulating protein biomarkers, gene expression does not require the generation of antibodies and the development of enzyme‐linked immunosorbent assays, which decrease the protein stability.^55^ Second, our study participants were recruited among women attending annual screening mammogram, which enhances generalizability.

Our study has several limitations. First, this is a cross‐sectional study and early life BMI (age 18, 30) and body shape at age 10 were self‐reported. However, recalled childhood adiposity at age 10 using Stunkard 9‐level figure somatotype pictogram and recalled early life BMI measurements (weight and height) have been validated.^56^, ^57^ Also, this study excluded participants whose pictograms were categorized as 8 and 9 since these were not derived in the Growing Up Today Study.^7^, ^29^

In conclusion, BMI changes from early life were associated with BMP2, RANK, RANKL, and TNFRSF13B gene expression in postmenopausal women. These findings offer new insights into potential mechanisms underlying the associations of adiposity and changes in adiposity during the life course with mammographic breast density in postmenopausal women and can be evaluated further within the context of breast cancer prevention.

## CONFLICT OF INTEREST

All authors declare no conflict of interest.

## AUTHOR CONTRIBUTIONS

Concept and design: Toriola. Acquisition, analysis, or interpretation of data: All authors. Drafting of the manuscript: Han. Critical revision of the manuscript for important intellectual content: All authors. Statistical analysis: Han. Obtained funding: Toriola. Administrative, technical, or material support: Toriola. Supervision: Colditz, Toriola.

## ETHICAL APPROVAL STATEMENT

This project was approved by the Ethical Committee of the Washington University School of Medicine, Saint Louis, Missouri, USA.

## Supporting information


Table S1–S2

## Data Availability

The data are available upon request from the corresponding author.
