# Supplementary material for: Changes in adiposity over the life course and gene expression in postmenopausal women
Source: Cancer Med. 2022 Mar 19;11(13):2699–710. doi: 10.1002/cam4.4649 (PMC9249983; doi:10.1002/cam4.4649)
Supplement: Supplementary file 1 — Table S1–S2 [file CAM4-11-2699-s001.docx]

| **Supplementary Table 1. Multivariable-adjusted associations between BMI at age 18 and plasma gene expression profile in 372 postmenopausal women^a^** | | |
| --- | --- | --- |
| **Genes^b^** | **Per 10 kg/m^2^ BMI increase at age 18** | |
|  | **Diff%^c^** | **95%CI** |
| **Growth factor-related genes** |  |  |
| **BMP2** | -7.3 | -19.3, 6.5 |
| **IGF1** | -4.1 | -19.0, 13.6 |
| **IGFBP3** | -1.5 | -11.8, 10.0 |
| **FGF1** | 2.1 | -3.3, 7.7 |
| **FGF12** | 1.6 | -3.8, 7.3 |
| **TGFB1** | 4.8 | -1.0, 10.9 |
| **RANK pathway-related genes** |  |  |
| **RANK** | -3.4 | -12.2, 6.3 |
| **RANKL** | -3.9 | -14.8, 8.4 |
| **TNFRSF13B** | -3.0 | -11.7, 6.6 |
| **TNFRSF18** | -0.5 | -6.2, 5.4 |
| **OPG** | -1.9 | -10.0, 6.9 |
| **Sex hormone-related genes** |  |  |
| **PRL** | -3.2 | -8.1, 1.9 |
| **PGR** | 3.4 | -4.9, 12.3 |
| **ESR1** | 2.5 | -4.0, 9.5 |
| **STAT1** | 2.9 | -6.1, 12.8 |
| **STAT5** | 0.7 | -5.1, 6.9 |
| Abbreviations: BMI, Body mass index; CI, Confidence Interval  ^a^ Multivariable-adjusted models were adjusted for age at mammogram (continuous, years), race (non-Hispanic white/African American/others), family history of breast cancer (yes/no/unknown), and menopausal hormone therapy (yes/no).  ^b^ Gene expression was presented as the mean and standard deviation of log_2_ transformed values.  ^c^ Diff% represents one-unit change in an adiposity measure associated with % change in gene expression. | | |

| **Supplementary Table 2. Multivariable-adjusted associations between BMI change from age 30 to age at mammogram and plasma genes expression profile in 372 postmenopausal women^a^** | | | | | | | |
| --- | --- | --- | --- | --- | --- | --- | --- |
| **Genes^b^** | **BMI change (continuous)** | **BMI change (categories)** | | | | | |
|  | **Per 10 kg/m^2^ BMI increase,**  **Diff% (95%CI)^c^** | **BMI loss,**  **Diff% (95%CI)^c^** | **BMI gain of 0.1-5 kg/m^2^,**  **Diff% (95%CI)^c^** | **BMI gain of 5.1-10 kg/m^2^,**  **Diff% (95%CI)^c^** | **BMI gain of 10.1-15 kg/m^2^,**  **Diff% (95%CI)^c^** | **BMI gain of >15 kg/m^2^,**  **Diff% (95%CI)^c^** | **P-trend** |
| **Growth factor-related genes** |  |  |  |  |  |  |  |
| **BMP2** | **16.2**  **(4.4, 29.4)** | -2.3  (-21.3, 21.3) | Ref | 10.2  (-5.6, 28.7) | **21.2**  **(0.2, 46.7)** | 28.2  (-0.8, 65.8) | **0.01** |
| **IGF1** | -0.4  (-12.7, 13.6) | -14.2  (-34.1, 11.8) | Ref | -15.8  (-30.3, 1.8) | -2.9  (-23.1, 22.6) | -8.6  (-33.2, 25.2) | 0.88 |
| **IGFBP3** | -2.6  (-10.6, 6.2) | 16.6  (-1.7, 38.4) | Ref | 9.9  (-2.8, 24.2) | -7.5  (-20.4, 7.6) | 7.7  (-12.1, 32.0) | 0.28 |
| **FGF1** | -1.3  (-5.4, 2.9) | 0.4  (-7.7, 9.2) | Ref | 1.4  (-4.5, 7.7) | -6.4  (-13.1, 0.8) | 5.1  (-4.9, 16.1) | **0.04** |
| **FGF12** | -0.8  (-4.9, 3.6) | -0.3  (-8.5, 8.6) | Ref | -2.4  (-8.2, 3.8) | -2.1  (-9.2, 5.6) | 1.5  (-8.4, 12.4) | 0.54 |
| **TGFB1** | -2.9  (-7.1, 1.5) | 2.6  (-6.1, 12.2) | Ref | 0.4  (-5.8, 7.0) | -3.0  (-10.4, 4.9) | -0.0  (-10.1, 11.1) | 0.62 |
| **RANK pathway-related genes** |  |  |  |  |  |  |  |
| **RANK** | -6.0  (-12.7, 1.3) | -0.2  (-14.0, 15.8) | Ref | -0.2  (-10.3, 11.0) | -4.7  (-16.4, 8.7) | -15.6  (-29.3, 0.7) | 0.053 |
| **RANKL** | **-12.6**  **(-20.3, -4.2)** | 1.9  (-15.4, 22.7) | Ref | -10.8  (-21.9, 1.9) | -9.8  (-23.4, 6.3) | **-26.0**  **(-40.6, -7.7)** | **0.005** |
| **TNFRSF13B** | **-10.5**  **(-16.8, -3.8)** | 12.2  (-3.0, 29.8) | Ref | -2.5  (-12.2, 8.2) | -11.3  (-21.9, 0.9) | -13.1  (-26.9, 3.3) | **0.003** |
| **TNFRSF18** | -2.7  (-7.0, 1.8) | 0.1  (-8.6, 9.7) | Ref | -2.2  (-8.4, 4.4) | -5.4  (-12.7, 2.5) | -5.0  (-14.8, 5.8) | 0.33 |
| **OPG** | -3.3  (-9.6, 3.4) | 2.1  (-10.8, 16.9) | Ref | -0.7  (-9.9, 9.4) | -0.7  (-11.8, 11.9) | -2.5  (-17.0, 14.4) | 0.62 |
| **Sex hormone-related genes** |  |  |  |  |  |  |  |
| **PRL** | 1.9  (-2.2, 6.0) | -2.5  (-10.1, 5.7) | Ref | 3.05  (-2.8, 9.2) | -1.7  (-8.4, 5.6) | 6.8  (-3.0, 17.6) | 0.09 |
| **PGR** | -4.8  (-10.7, 1.6) | 0.1  (-12.2, 14.0) | Ref | -2.37  (-11.1, 7.2) | -2.1  (-12.7, 9.8) | -8.2  (-21.4, 7.2) | 0.29 |
| **ESR1** | -2.2  (-7.1, 2.9) | -1.3  (-10.9, 9.4) | Ref | -2.34  (-9.3, 5.1) | -0.6  (-9.2, 8.8) | -6.7  (-17.4, 5.4) | 0.41 |
| **STAT1** | -4.1  (-10.7, 2.9) | 5.3  (-8.7, 21.5) | Ref | 2.06  (-7.9, 13.1) | -2.4  (-13.9, 10.7) | -7.4  (-21.9, 9.7) | 0.55 |
| **STAT5** | 0.6  (-3.9, 5.4) | 0.0  (-8.8, 9.7) | Ref | 6.5  (-0.3, 13.8) | -3.0  (-10.6, 5.2) | 4.3  (-6.5, 16.4) | 0.13 |
| Abbreviations: BMI, Body mass index; CI, Confidence Interval  ^a^ Multivariable-adjusted models were adjusted for age at mammogram (continuous, years), BMI at age 10 (continuous, kg/m^2^), race (non-Hispanic white/African American/others), family history of breast cancer (yes/no/unknown), and menopausal hormone therapy (yes/no).  ^b^ Gene expression was presented as the mean and standard deviation of log_2_ transformed values.  ^c^ Diff% represents one-unit change in an adiposity measure associated with % change in gene expression. | | | | | | | |
